# Spatial Virome Analysis of *Zanthoxylum armatum* Trees Affected With the Flower Yellowing Disease

**DOI:** 10.3389/fmicb.2021.702210

**Published:** 2021-06-28

**Authors:** Mengji Cao, Song Zhang, Ruiling Liao, Xiaoru Wang, Zhiyou Xuan, Binhui Zhan, Zhiqi Li, Jie Zhang, Xinnian Du, Zhengsen Tang, Shifang Li, Yan Zhou

**Affiliations:** ^1^National Citrus Engineering Research Center, Citrus Research Institute, Southwest University, Chongqing, China; ^2^State Key Laboratory of Biology for Plant Diseases and Insect Pests, Institute of Plant Protection, Chinese Academy of Agricultural Sciences, Beijing, China; ^3^Jiangjin Agricultural Technology Extension Station, Chongqing, China; ^4^Bishan Modern Agricultural Development Promotion Center, Chongqing, China; ^5^Zhaotong Forestry and Grassland Pest Monitoring and Testing Center, Yunnan, China; ^6^Environment and Plant Protection Institute, Chinese Academy of Tropical Agricultural Sciences, Haikou, China

**Keywords:** *Zanthoxylum* viruses, *Ilarvirus*, satellite RNA, RNA-seq, small RNAs

## Abstract

*Zanthoxylum armatum* is an important woody crop with multiple applications in pharmaceutics, cosmetics, and food industries. With continuous increases in the plantation area, integrated pest management is required for scale production when diseases caused by biotic factors such as pests and pathogens have become new problems, one of which is the infectious flower yellowing disease (FYD). Here, isolates of a new illarvirus (3) and a new nepovirus-associated subviral satellite RNA (12) were identified in *Z. armatum*, in addition to 38 new isolates of four previously reported RNA viruses. Sequence variation can be observed in viral/subviral quasispecies and among predominant isolates from the same or different samples and geographic origins. Intriguingly, RNA sequencing of different diseased trees invariably showed an extraordinary pattern of particularly high reads accumulation of the green Sichuan pepper-nepovirus (GSPNeV) and the satellite RNA in symptomatic tissues. In addition, we also examined small RNAs of the satellite RNA, which show similar patterns to those of coinfecting viruses. This study provides further evidence to support association of the FYD with viral/subviral infections and deepens our understanding of the diversity and molecular characteristics of the viruses and satellite, as well as their interactions with the host.

## Introduction

*Zanthoxylum*, one of the most economically important genera in the family Rutaceae, comprises deciduous, spiny shrub species (Ekka et al., 2020). Some species, the so-called Chinese prickly ash, are well-known in China, especially in southwestern regions, for large-scale cultivation for production of spices, medicines, and essential/edible oils (Zhang et al., 2017; Ma et al., 2020). *Zanthoxylum armatum* (Zhuye huajiao in Chinese), also known as green Sichuan (Szechwan) pepper, is a commercial species that generates green fruits that can be used as a seasoning for its special aroma and numbing flavor (Xu et al., 2019). There is one representative production area and trade center in Chongqing Municipality, that is, Jiangjin District, where *Z. armatum* var. *novemfolius* as a native cultivar with an annual output value of nearly half a billion dollars has been widely planted. *Z. armatum* orchards are generally monocultural with one or few commercial cultivars planted locally, which, despite convenient to manage, are prone to be affected by disease outbreaks. In recent years, a virus-like disease, the flower yellowing disease (FYD), that leads to a severe disorder of the floral organs, has emerged as the main restriction factor for the industrial development of *Z. armatum* (Zhang et al., 2020). The FYD is typically characterized by symptoms of pistil abortion, stamen yellowing and intumescence, usually with yellowing and stunting of the foliage, so as to be extremely destructive to fructification of the affected plants (Cao et al., 2019). This disease may occur first on a few branches and then extends progressively to the whole plant, causing irreversible tree decline and eventually death, thus resulting in huge economic losses. Similar problems also arise in other *Zanthoxylum* species, including *Z. bungeanum*. However, Koch’s postulates are yet to be fulfilled to explain the etiology of whether a pathogen is involved.

With its unbias and high sensitivity, high-throughput sequencing (HTS) has been extensively applied for identification and detection of viruses present in various organisms, such as the research on all plant viruses in samples from single or multiple plant species under specific conditions (Coetzee et al., 2010; Massart et al., 2014; Bernardo et al., 2018; Maclot et al., 2020). The global plant virome has revealed extensive viral diversity, allowing us to gain insight into the roles these viruses play in ecosystems across scales, from simple cytozoic parasites to the essential impetus of plant evolution (Dolja et al., 2020). For instance, plant viruses can easily have adverse effects on agroecosystem due to their pathogenicity to sensitive hosts, and there is an epidemic risk in genetically uniform crops in the presence of biological vectors as vehicles (Gilbertson et al., 2015).

With the aid of HTS, we have previously shown diverse viruses in *Zanthoxylum* species that are transmissible among trees in agricultural environments and possibly pathogenic, and thus they are potential threats to the sustainability of the industry (Cao et al., 2019). The FYD is possibly caused by a nepovirus, green Sichuan pepper-nepovirus (GSPNeV), which is frequently accompanied by one or several other viruses in the plant. It seems that GSPNeV is present mainly in symptomatic branches in a tree before it disturbs asymptomatic branches and causes symptoms, based on the results of reverse-transcription polymerase chain reaction (RT-PCR). In the present work, this phenomenon was further studied by HTS analysis, and this showed a large number of reads derived from GSPNeV in symptomatic branches but very few reads in asymptomatic branches, thus supporting an uneven viral/subviral distribution and likely suggesting its association with the disease pattern of non-uniform symptoms. Moreover, a deep analysis regarding co-evolution and co-transmission of the viral/subviral RNAs and the dynamics of their sequence variation was also conducted.

## Materials and Methods

### Sample Collection

A survey was first conducted in orchards to identify *Zanthoxylum* trees affected by the flower yellowing disease (FYD) with symptomatic and asymptomatic branches on a single plant. Six samples (foliar and floral tissues mixed for each) from symptomatic and asymptomatic branches of three selected partial diseased *Z. armatum* trees, one from Yunnan Province and two from Chongqing Municipality, were collected for RNA sequencing (RNA-seq) and subsequent comparative analyses of viral/subviral species and accumulation. In addition, 14 samples, each from a completely diseased tree or tree without symptoms as a negative control were incorporated for viral/subviral diversity and variation analyses. In total, 20 samples were separately sequenced for etiology study of the FYD, in addition to a diseased tree (marked here as CQ1-D) with RNA-seq data from a previous study (Cao et al., 2019). Among these 21 samples, six were from Zhaotong City in Yunnan (YN) Province (YN1-DS, YN1-DA, YN2-D, YN3-H, YN4-H, YN5-H), while the others were from Chongqing (CQ) Municipality, one in Bishan District (CQ4-D), two in Jiangjin District (CQ1-D and CQ2-D), five in Tongnan District (CQ3-H, CQ5-D, CQ6-H, CQ7-DS, CQ7-DA), and seven in Changshou District (CQ8-D, CQ9-D, CQ10-D, CQ11-D, CQ12-H, CQ13-DS, CQ13-DA). Among these, symptomatic samples from completely diseased trees were indicated with D (diseased), symptomatic samples from partial diseased trees were indicated with DS (diseased-symptomatic) while from the same trees asymptomatic samples indicated with DA (diseased-asymptomatic), and samples from trees without any symptoms were tentatively marked with H (healthy). The sample information is shown in Table 1.

**TABLE 1:** Sampling information, sequencing data size, and proportion (%) of viral/subviral RNA reads in total reads.

Sample	^a^YN1-DS	YN1-DA	YN2-D	YN3-H	YN4-H	YN5-H	CQ1-D	CQ2-D	CQ3-H	CQ4-D	CQ5-D	CQ6-H	CQ7-DS	CQ7-DA
					
Location	^b^ZT						JJ		TN	BS	TN			
SRA accessions	SRR14663392	SRR14663391	SRR14663380	SRR14663378	SRR14663377	SRR14663376	SRR14663375	SRR14663374	SRR14663373	SRR14663372	SRR14663390	SRR14663389	SRR14663388	SRR14663387
Total reads	61,008,212	60,539,870	67,129,108	64,411,510	76,455,572	56,140,062	58,077,354	57,580,796	56,724,560	53,849,114	54,642,862	57,458,864	49,756,462	64,684,982
GSPNeV_RNA1	8.32%	0	4.46%	0	0	0	3.16%	4.53%	0	5.75%	4.30%	0	3.69%e	0
GSPNeV_RNA2	9.24%	0	4.39%	0	0	0	4.02%	5.14%	0	8.62%	7.33%	0	5.89%	0
SatGSPNeV	13.34%	0	3.62%	0	0	0	8.91%	8.76%	0	20.33%	13.42%	0	11.72%	0
GSPIdV_RNA1	0.21%	0	0.006%	0	0	0.007%	1.57%	0.7%	0	0.95%	1.72%	0	2.89%	1.1%
GSPIdV_RNA2	0.14%	0	0.02%	0	0	0.05%	3.73%	2.49%	0	2.19%	4.64%	0	3.49%	2.91%
GSPNuV_RNA	0	0	0	0	0	0	0.06%	0	0	0	0	0	0	0
GSPEV_RNA	0	0	0.94%	0.004%	0.005%	0.07%	0.4%	0.33%	0.60%	0.03%	0.01%	0.06%	0	0.01%
GSPIlV_RNA1	0.08%	0	0.09%	0	0	0	0	0	0	0	0	0	0.73%	0
GSPIlV_RNA2	0.07%	0	0.08%	0	0	0	0	0	0	0	0	0	0.7%	0
GSPIlV_RNA3	0.28%	0	0.42%	0	0	0	0	0	0	0	0	0	3.74%	0.0008%

**Sample**	**CQ8-D**	**CQ9-D**	**CQ10-D**	**CQ11-D**	**CQ12-H**	**CQ13-DS**	**CQ13-DA**							
		
**Location**	**CS**													

SRA accessions	SRR14663386	SRR14663385	SRR14663384	SRR14663383	SRR14663382	SRR14663381	SRR14663379							
Total reads	68,344,842	62,910,592	70,511,028	80,552,614	58,534,686	71,598,316	84,446,876							
GSPNeV_RNA1	7.12%	5.47%	5.3%	5.32%	0	6.36%	0							
GSPNeV_RNA2	12.74%	9.61%	11.8%	9.91%	0	12.4%	0.0002%							
SatGSPNeV	12.12%	18.35%	16.55%	0	16.89%	0.0002%								
GSPIdV_RNA1	0.52%	0.9%	1.6%	0.69%	0	1.45%	9.52%							
GSPIdV_RNA2	1.02%	2.7%	3.22%	2.02%	0	6.05%	1.11%							
GSPNuV_RNA	0	0	0	0	0	0	0							
GSPEV_RNA	6.36%	2.2%	6.04%	6.62%	1.09%	0.35%	0.21%							
GSPIlV_RNA1	0	0	0	0	0	0	0							
GSPIlV_RNA2	0	0	0	0	0	0	0							
GSPIlV_RNA3	0	0	0	0	0	0	0							

*^a^Yunnan (YN) Province, Chongqing (CQ) Municipality; D, symptomatic sample from diseased (D) tree with systematic symptoms; DS, symptomatic (S) sample from diseased (D) tree with partial symptoms; DA, asymptomatic (A) sample from the same diseased (D) tree with partial symptoms; H, sample from tree without symptoms that appears to be healthy (H). ^b^Zhaotong (ZT) City, Jiangjin (JJ) District, Tongnan (TN) District, Bishan (BS) District, Changshou (CS) District.*

### High-Throughput Sequencing and Data Processing

The collected tissues of each sample were used for total RNA extraction by the EASY spin Plus Complex Plant RNA Kit (Aidlab, Beijing, China). The RNA was tested using a Nanodrop (Thermo Fisher Scientific, Waltham, United States) and agarose-gel electrophoresis to ensure a high quality. After ribosome RNA depletion by the RiboZero Magnetic Kit (Epicenter, Madison, United States), a library was built with the TruSeq RNA Sample Prep Kit (Illumina, San Diego, United States). The treated RNA was then sequenced by the Mega Genomics Company (Beijing, China) using an Illumina HiSeq X-ten platform with 150 bp layout in paired-end read length. Total small RNA (sRNA) was extracted with the EASYspin Plant microRNA Extract Kit (Aidlab, Beijing, China), constructed as a library using the TruSeq Small RNA Sample Prep Kit (Illumina) and sequenced on an Illumina Hiseq2500 platform with a length of 50 bp per reading (Mega Genomics). A series of built-in programs in the CLC Genomics Workbench 11 (Qiagen, Hilden, Germany) were employed to process generated RNA-seq and sRNA-seq raw data in the following steps: (1) remove adaptors and low-quality reads, and the host-related reads by mapping to draft genomes of citrus within Rutaceae family (*Zanthoxylum* genomes are publicly unavailable) used as references (Xu et al., 2013); (2) *de novo* assemble the remaining reads into contigs using *De Novo* Assembly program with default parameters of *de Bruijn* graph word size 20, and minimum contig length 200 bp; and (3) annotate the contigs using a local BLASTx program with the viral sequence database (taxid:10239) downloaded from the National Center for Biotechnology Information (NCBI) used as a target.

### Verification of Viral/Subviral Sequence

To exclude the possibility of chimeric viral/subviral sequences that result from assembly program algorithm error and false virus-positive results caused by contamination during HTS, contigs of the new ilarvirus from CQ7-DS and the satellite from CQ1-D were verified by RT-PCR using the leaf RNA extract, viral/subviral contig-specific primers (Supplementary Table 1), and a One-step RT-PCR Kit (Takara, Otsu, Japan). Specifically, the terminal sequences of the satellite were obtained using a commercial RACE Kit (Invitrogen, Carlsbad, United States). The amplicons were purified through the Gel Extraction Kit (Omega Bio-Tek, Norcross, United States), cloned with the pEasy-T1 Vector System (TransGen, Beijing, China), and sequenced by the TsingKe company (Beijing, China)—five clones per viral/subviral PCR amplicon. The resulting sequences were merged using the SeqMan (DNASTAR, Madison, United States), and assembled sequences were submitted to NCBI-GenBank and assigned specific accession numbers (see section “Data Availability Statement”). Subsequently, these sequences and available sequences from databases were used as reference genomes, onto which the corresponded viral/subviral reads of each sequencing data were mapped to obtain consensus sequences between mapped reads and the referenced sequences. The consensus sequences were compared with corresponding viral/subviral contigs generated from the assembly of independent reads of all data to ensure sequence fidelity, and were also submitted to GenBank. This comparison analysis was aimed at eliminating the interference of viral/subviral variation and recombination with the assembly process; it requires a high nt identity (99%) between two sequences; if not satisfied, it is necessary to confirm the consensus sequence using molecular cloning and sequencing.

### Virome and Sequence Analyses

A heatmap of the read numbers of viral/subviral RNAs in different samples (clustering by the squared Euclidean distance) was drawn by Heat Map with Dendrogram extension in Origin 2017 software (OriginLab, Northampton, United States), and a flower plot of common (center) or unique (petals) viral/subviral RNAs across symptomatic or symptomatic/healthy samples was drawn by R (ver. 4.0.2) using draw.ellipse and draw.circle functions according to previous research (Sugawara et al., 2013). Open reading frames (ORFs) in viral/subviral sequences were predicted using the NCBI ORF finder^1^, and conserved protein domains were searched with the Conserved Domain Database (CDD) web tool^2^. Sequence comparison was conducted in the CLC Genomics Workbench 11 to identify conserved sites among viral/subviral sequences.

### Phylogenic and Population Analyses

Viral/subviral nucleotide (nt) and amino acid (aa) sequences from this study and the GenBank databank were aligned using MAFFT 7 (Katoh and Standley, 2013); poorly aligned regions were trimmed using trimAl (Capella-Gutiérrez et al., 2009), and the remaining regions were imported into MEGA-X (Kumar et al., 2018) to select the best-suited model under the Bayesian information criterion. The maximum-likelihood phylogenetic relationships (500 bootstrap replications) were inferred, and the four rate categories of a discrete Gamma distribution were applied if necessary (Yang, 1996). RDP4 (Martin et al., 2015), SDT 1.2 (Muhire et al., 2014), the default Alignments tool, and Low Variant Detectors in the CLC Genomics Workbench 11 were used in viral/subviral population comparison for recombination, sequence identity matrix, sequence alignment (the identity plot), and variation analysis (the latter two were used to study evolutionary hotspots), respectively. A histogram of the sequence variation was visualized with the ggplot2 package 3.3.2 in R (Wickham, 2011). A variation heatmap of quasispecies was drawn with the pheatmap package 1.0.12 in R (Kolde and Kolde, 2015). The network was drawn by Cytoscape 3.8.0 to indicate virus phylogenetic-geographical relationships (Shannon et al., 2003), where greater phylogenetic incongruity between two viral/subviral RNAs and their more specific geographic origins increased the edge thickness of their nodes (a thick line indicates high confidence of viral/subviral RNA co-evolution or co-transmission), and an attribute circle layout was adopted. The penalty rules used to indicate the thickness are as follows: (i) default penalty between two nodes = 1; (ii) for one more phylogenetic incongruity or specific geographic origin, the penalty is + 1; (iii) the lower the penalty, the greater the thickness.

### Small RNA Analysis

The small RNA data previously used were reanalyzed to extract sRNA characteristics of the new satellite RNA that is likely associated with the nepovirus (Cao et al., 2019). Reads were mapped to the satellite sequence using the CLC Genomics Workbench 11. Size distribution and the 5′-nucleotide (5′-nt) preference of satellite sRNAs were counted and schematized using Origin 2017. The distribution of sRNAs in the positive (pos) and negative (neg) strands of the satellite RNA was visualized by the ggplot2 package in R. For sRNA size distribution of the satellite, the Kolmogorov–Smirnov (K–S) method in Origin 2017 was used to test the normality, and then the distribution was fitted with a Gaussian function. The viral/subviral RNA-clustering sRNA size and 5′-nt heatmaps were Z-score normalized and plotted using the heatmap.2 function (gplots package 3.1.0) in R (Warnes et al., 2016).

## Results

### Virus Identification and Sequence Confirmation

After data processing, a total of 49,756,462 (6.95 Gb)–84,446,876 (11.8 Gb) clean reads were obtained for individual sequencing of 20 samples except that of CQ1-D, which was previously published (Table 1). The reads of each sample were assembled and annotated independently to detect known viruses and any potential new viruses. This allowed identification of contigs related to the known green Sichuan pepper-nepovirus (GSPNeV), -idaeovirus (GSPIdV, note that its abbreviation in previous study is GSPIV), -enamovirus (GSPEV), and -nucleorhabdovirus (GSPNuV), and the new contigs homologous to the *Nepovirus* genus-associated satellite RNA and viruses in the genus *Ilarvirus* (family *Bromoviridae*), based on the *e*-values (> 5e–04 for the former, and > 6e–98 for the latter) from BLASTx analysis. After verification by RT-PCR, cloning, and sequencing of complete nucleotide sequences of the satellite and most of the ilarvirus genome and based on preliminarily taxonomic analyses, we tentatively named the satellite and ilarvirus “green Sichuan pepper-nepovirus large satellite RNA” (satGSPNeV) and “-ilarvirus” (GSPIlV), respectively. With the use of GSPNeV, satGSPNeV, GSPIdV, and GSPEV from the CQ1-D sample and GSPIlV from the CQ7-DS sample as reference genomes for read mapping, viral/subviral consensus sequences between the references and mapped reads from other samples were obtained. Since viral/subviral nucleotide sequences derived from independent assembly of all sample data were highly identical to the consensus sequences (>99%), these consensus sequences were considered predominant and accurate and were directly used for other analyses. If high sequence heterogeneity is present in a viral/subviral quasispecies that can affect sequence assembly, contigs from assembly and consensus sequences from read mapping with different parameters (threshold values: similarity = 0.4–0.8, fraction = 0.4–0.8) will be obviously distinct from one another, and our tests showed that the viral/subviral sequences resulting from different conditions were highly consistent.

### Viral/Subviral Constituents and Accumulation in Samples

**FIGURE 1:**
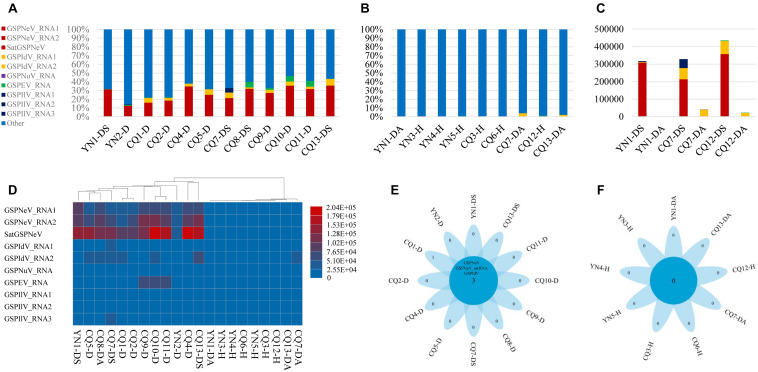
Virome analysis of *Z. armatum* trees affected with the flower yellowing disease and the trees without obvious symptoms. Proportion of reads associated with the viral and subviural RNAs, relative to total reads of each symptomatic sample **(A)**, and asymptomatic/healthy sample **(B)**, and reads of evolution-associated RNAs, i.e., two RNAs of green Sichuan pepper-nepovirus (GSPNeV) with green Sichuan pepper-nepovirus large linear satellite RNA (satGSPNeV), two RNAs of green Sichuan pepper-idaeovirus (GSPIdV) or three RNAs of green Sichuan pepper-ilarvirus (GSPIlV), were counted together. Viral/subviral read number comparison between symptomatic and asymptomatic branch samples from the same tree, with three independent partial diseased trees analyzed **(C)**. In figures **(A–C)**, the colors of bars indicate different viruses, and satGSPNeV is shown the same color with GSPNeV. Heatmap of read numbers of viral and subviral RNAs from different samples, with the clustering of samples **(D)**; the deeper the colors of bars (from blue to red), the higher the numbers of reads specific to each viral/subviral RNA. Flower plots indicate the numbers of common virus/satellite across the symptomatic **(E)** or asymptomatic/healthy **(F)** samples that are shown in the central blue circles or of specific virus/satellite in each sample that are shown in the petals; two viruses and one satellite were detected in all symptomatic samples that have been marked with D or DS, but no common virus/satellite was detected in asymptomatic/healthy samples that have been marked with DA or H. Green Sichuan pepper-enamovirus (GSPEV), and -nucleorhabdovirus (GSPNuV).

Overall, reads from GSPNeV and satGSPNeV were more abundant in diseased samples, in which they accounted for 12–36% of the total reads relative to the reads from all other viruses (<11% for GSPIdV, GSPEV, GSPNuV, GSPIlV), but in those samples for which symptoms were not obvious, the read proportions for GSPNeV and satGSPNeV were < 0.003% (Figures 1A,B and Table 1). A more specific comparison of three biological repeats (Figure 1C and Table 1) between symptomatic and asymptomatic branch samples from the same trees showed a striking accumulation of GSPNeV and satGSPNeV rather than other viruses in symptomatic samples, not in asymptomatic samples (almost no reads), and this result was confirmed by RT-PCR: GSPNeV and satGSPNeV were only detected in symptomatic samples. Symptomatic and asymptomatic samples were independently clustered by read numbers of viral/subviral RNAs (Figure 1D), indicating a special pattern of viral/subviral infections in symptomatic samples, where GSPNeV and satGSPNeV were major factors while other viruses were minor, because although GSPIlV occurred in all diseased trees, it was also found in asymptomatic trees with a moderate read abundance (Figures 1E,F and Table 1). Correlations of GSPNeV with satGSPNeV and the FYD shown by HTS analyses were confirmed by RT-PCR and gel electrophoresis (Supplementary Figure 1). Collectively, these data suggested GSPNeV and satGSPNeV are closely associated with the FYD across two type of spaces, namely, different branches of a single tree and different tree geographical positions.

### Sequence and Phylogenetic Analyses

**FIGURE 2:**
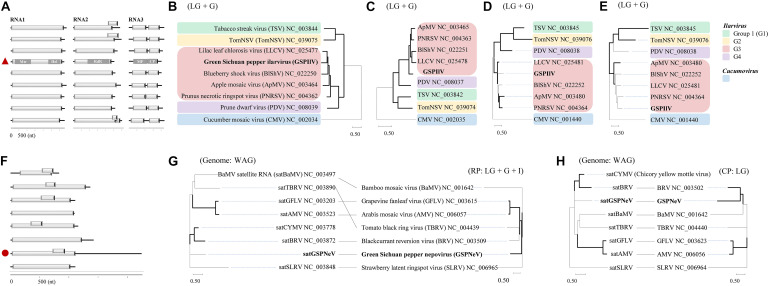
Sequence and phylogenetic characteristics of green Sichuan pepper-ilarvirus (GSPIlV) and green Sichuan pepper-nepovirus large linear satellite RNA (satGSPNeV). Genomic comparisons of GSPIlV (the red triangle) and ilarviruses **(A)**. Mtr, methyltransferase; Hel, helicase; MP, movement protein; CP, coat protein. Phylogenetic relationships of GSPIlV and ilarviruses inferred from alignments of replicase (RP, **B**), RNA-dependent RNA polymerase **(C)**, MP **(D)**, and CP **(E)** sequences; cucumber mosaic virus (CMV) was used as an outgroup. RNA structure of satGSPNeV (**F**, the red circle). Phylogenetic trees and co-evolution analyses of satellite RNAs (**G,H**, left) and their helper viruses (**G,H**, right) based on satellite nucleotide sequences and helper virus RPs (**G**, right) and (**H**, right). The models in MEGA-X selected for phylogenies are indicated. The nodes with > 50% bootstrap supports are shown by thick lines.

The GSPIlV from the CQ7-DS sample (viral isolate IlCQ7-DS; isolate name = the first two letters of the virus genus name or the “satellite” plus the sample name) was partially sequenced, and its nearly complete genomes are tripartite, exhibiting canonical genomic organizations found in the genus *Ilarvirus*, especially the subgroup III (G3) members (Noda et al., 2017; Bratsch et al., 2019), that is, typical monocistronic RNA1, RNA2, and bicistronic RNA3 (Figure 2A). Extensive identical sequences were observed at the 3′ genomic ends (Supplementary Figure 2). RNA1 (∼ 3.4 kb) contains a large ORF1 (nt 37–3,150), potentially coding for a replicase (Rep, 1,037 aa) based on methyltransferase (Mtr, aa 51–387, pfam01660) and helicase (Hel, aa 745–1,002, pfam01443) domains detected by CDD search. RNA2 (∼ 2.3 kb) with ORF2 encodes a putative protein (765 aa) with an RNA-dependent RNA polymerase (RdRp, aa 271–707, pfam00978) domain. RNA3 harbors two ORFs, ORF3a (nt 170–994) and ORF3b (nt 998–1,696), which were predicted to encode movement protein (MP, 274 aa; domain cl03270 at aa 7–262), and coat protein (CP, 232 aa; domain cl03355 at 29–227), respectively. In BALSTx analyses based on the NCBI Viruses database (taxid:10239), GSPIlV showed greater sequence homology to ilarviruses belonging to G3 species (score rank < 40), compared with other viruses (score rank > 40). We conducted sequence comparison analysis and found evidently higher nt/aa sequence identities that GSPIlV shared with G3 species than with others (Supplementary Figure 3). In addition, GSPIlV was phylogenetically closest to the G3 ilarviruses, independent of the proteins used for the analyses (Figures 2B–E). We propose that GSPIlV could be a member of a new species in G3 of the genus *Ilarvirus*.

The full-length sequence of the satGSPNeV isolate from sample CQ1-D (subviral isolate SaCQ1-D) was obtained, which comprises 2,247 nts excluding the poly(A) tail. Its genomic 5′ and 3′ termini are to some extent conserved compared with GSPNeV RNAs (Supplementary Figure 4). Two ORFs, one (nt 713–928) encompassed in the other (nt 13–1,110), were predicted in the sequence (Figure 2F), but the putative protein products (365-aa and 71-aa, respectively) from them were functionally unknown. Interestingly, compared with other related satellite RNAs associated with other viruses, satGSPNeV has an extraordinary long 3′ untranslated region (1,141-nt), similar to two genomic RNAs of GSPNeV. The nt sequence identity shared with other viral satellite RNAs was not significant (< 29%), in addition to the aa sequence identity for the larger protein (< 23%). Despite this, BLASTp analysis of this protein suggested that it is homologous to those of blackcurrant reversion virus satellite RNA (*e* = 1.85E–10) and chicory yellow mottle virus large satellite RNA (*e* = 8.71E–23). The phylogenetic analysis with the complete nucleotide sequences also placed satGSPNeV with these two satellite RNAs (Figures 2G,H, on left). When related nepoviral RNA1 and RNA2 polyproteins were additionally included for co-evolution analysis, more phylogenetic incongruities were observed between Rep-encoding RNA1-polyprotein and the satellites (Figures 2G,H, on right); therefore, it appears that the coat protein-encoding RNA2s of their helper viruses are more evolutionarily associated with them. In fact, the other satellite RNAs analyzed may have a CP origin due to their close phylogenetic relationships with some viral CP (Alazem and Lin, 2017). However, this hypothesis does not have any molecular support. Based on this evidence, satGSPNeV should be a new *Nepovirus*-associated large satellite RNA.

### Phylo-Genetic-Geographic Analysis

**FIGURE 3:**
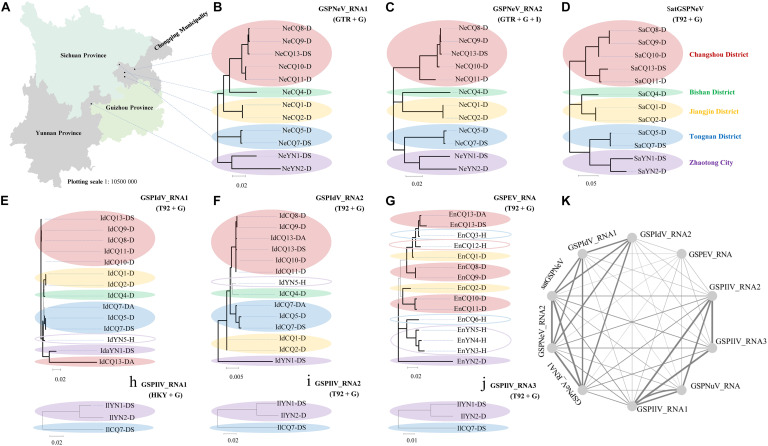
Phylogenetic and geographical analysis of available viral/subviral isolates. A map showing the sampling sites, which are shown with black circles **(A)**. Co-evolution and co-transmission relationship constructions of green Sichuan pepper-nepovirus—GSPNeV **(B,C)**, -nepovirus large linear satellite RNA—satGSPNeV **(D)**, -idaeovirus—GSPIdV **(E–G)**, -enamovirus—GSPEV **(H)**, -nucleorhabdovirus—GSPNuV (analysis not available for only one isolate), and -ilarvirus—GSPIlV **(H–J)**, based on the phylogenetic and geographical differences. The tree structures and geographical distributions of GSPNeV RNAs and satGSPNeV were used as references for comparisons and penalty point calculations. Isolates with the same locations are indicated by solid ellipses with the same color, and hollow ellipses represent isolates with unique distributions, compared with GSPNeV RNAs and satGSPNeV. Network visualization of the relationships by adding up penalties between two RNAs **(K)**; each node represents a different RNA. The models recommended by MEGA-X for phylogenetic analyses are indicated. The nodes with significant bootstrap values (>50%) are highlighted with thick lines.

Based on the viral/subviral consensus sequences (or preponderate sequences) of quasispecies obtained from the read mapping of available samples, phylogenetic dendrograms of the viral/subviral RNAs from different isolates were constructed to gain insight into their co-evolution (single viral-species/satellite) and co-transmission (two more viral-species/satellite) relationships by weighting both phylogenetic and geographic discrepancies between the trees (Figure 3). The populations from five independent locations were analyzed (Figure 3A). For GSPNeV RNAs and satGSPNeV, the consensus sequences from different locations were, in a similar phylogenetic architecture, clearly separately clustered by the locations (Figures 3B,C), thus suggesting their strong associations and simple population structures without noteworthy long-distance movements and cross infections. A lower degree of association was observed between GSPIdV RNAs or among GSPIdV, GSPNeV RNAs, and satGSPNeV (Figures 3B–F), probably because of genomic reassortments or mixed infections of GSPIdV. In contrast, the evolutionary statuses of GSPEV were complicated in contrast to these viruses and the satellite (Figure 3G), where there are likely to be independent genetic and geographic development trajectories since the phylogenetic clustering and range of infected samples were largely distinct. GSPIlV also displayed population separation by geographic positions in spite of a small number of infected samples available (Figures 3H–J). However, the much smaller number of sequenced samples (only one) that GSPNuV infected made analysis impossible. The results resembling those of the phylogenetic analyses were obtained from the sequence identity matrixes, where the 0–14.2% nt differences were shown between the consensus sequences of different viral/subviral RNAs (Supplementary Figure 5). Finally, by summing the penalties from phylogenetic and geographic incompatibility, a network was built that formed two main clustering groups, one for GSPNeV, GSPIdV RNAs, and satGSPNeV, and another for GSPIlV RNAs and GSPNuV, and a distant node for GSPEV, signifying the presence of both historically evolutionary similarities and differences among the viruses and satellite (Figure 3K).

### Sequence Variation Site Pattern and Recombination Analyses

**FIGURE 4:**
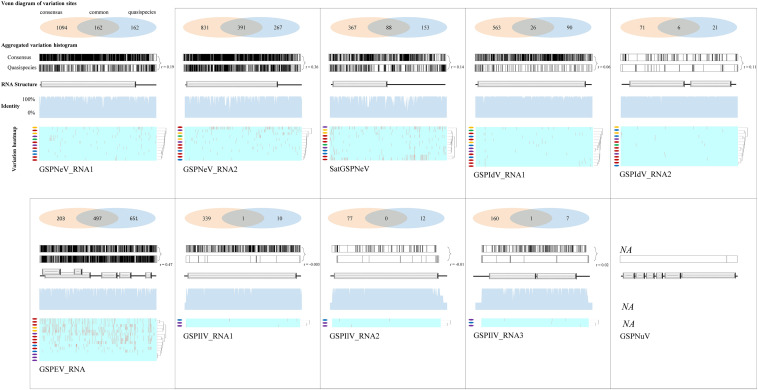
Variation sites pattern analyses of viral/subviral RNAs. Green Sichuan pepper-nepovirus (GSPNeV), -idaeovirus (GSPIdV), -enamovirus (GSPEV), -nucleorhabdovirus (GSPNuV), -nepovirus large linear satellite RNA (satGSPNeV), and -ilarvirus (GSPIlV). For each RNA, as each diagram shows, variation sites among the consensus sequences from the read mapping of different samples to reference isolates from CQ1-D and CQ7-DS or aggregated from comparisons of each consensus sequence with their own quasispecies were calculated (the Venn diagram) and visualized (the variation histogram). The common variation sites between the consensus sequences and quasispecies were also determined, and the counted numbers shown for the consensuses and quasispecies are unique. The plots for sequence identity among the consensus sequences are shown (the identity histogram). The variation heatmap at the bottom shows the clustering of the samples by variation site distributions of their quasispecies, and the quasispecies from the same geographical locations are indicated with the same colored circles.

Viral/subviral isolates from CQ1-D and CQ7-DS samples were used as reference genomes, based on which nucleotide sequence variation sites from comparisons with other available conceptional isolates (the consensus sequences) were counted and added together (Figure 4, the histograms of each graphical illustration), regardless of definite variation type (AUCG) or length (insertions and deletions). Within-quasispecies variations were also analyzed, using the viral/subviral isolates from each sample as a reference (Figure 4, below the consensus), as well as the Spearman correlation coefficient (r) between the consensus sequences and quasispecies variations. Generally, the range of viral/subviral variation sites from the consensus sequences was larger than that of the quasispecies, and the only exception was GSPEV, which suggests that it has a relatively faster variation speed under similar environmental conditions. Regardless, the r for variation site comparisons of the consensus sequences and quasispecies was low (< 0.5), which, besides a smaller size of the genetic pool in quasispecies, is also potentially due to different natural selections specific to each quasispecies before a structurally stable population. Even so, Spearman correlation analysis regarding trends in the numbers of variation sites from the consensus sequences, quasispecies, and their common variation sites (Figure 4, the Venn diagrams) showed that these items of different viral/subviral RNAs had strong correlations, with *r* > 0.77 (Supplementary Figure 6). With increasing numbers, the genome-wide distribution of variation sites from both the consensuses and quasispecies became more even, but sequence identity analyses suggested the presence of some evolutionary hotspots in the viral/subviral RNAs (Figure 4). Thus, there was great variation complexity in some regionally specific sites. Variation site heatmaps of the RNAs from different quasispecies showed that the different isolates were sometimes grouped by geographic positions, but most were irregularly clustered (Figure 4). Viral sequence variation is generally affected by multifactors other than single factors (García-Arenal et al., 2001; Domingo and Perales, 2019), such as environments and hosts, let alone different stages of viral populations; therefore, these clusters are not abnormal. Recombination analyses were also performed for the available consensus sequences of the viruses (Table 2), and the results showed that GSPNeV/satGSPNeV and GSPEV with more variation sites in the quasispecies had more recombination events based on the program supports (> 2) and threshold *e*-values (< E–5).

**TABLE 2:** Recombination events prediction for the viral/subviral RNAs.

Viral/subviral sequence	Significant event	Lowest method support (level < E–5)
GSPNeV_RNA1	9	2
GSPNeV_RNA2	4	2
SatGSPNeV	5	3
GSPIdV_RNA1	0	NA
GSPIdV_RNA2	0	NA
GSPNuV_RNA	NA	NA
GSPEV_RNA	9	2
GSPIlV_RNA1	0	NA
GSPIlV_RNA2	0	NA
GSPIlV_RNA3	0	NA

### Small RNA Profiles of the Satellite

**FIGURE 5:**
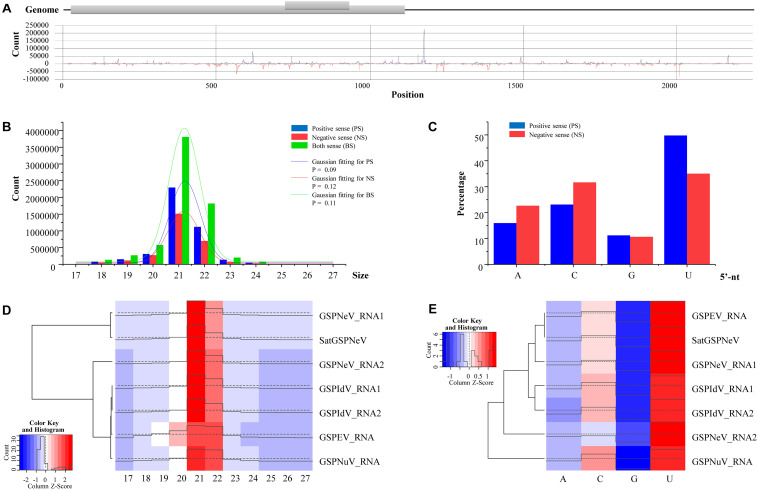
Small RNA (sRNA) analysis of green Sichuan pepper-nepovirus large linear satellite RNA (satGSPNeV). Distribution of sRNAs derived from positive and negative strands of satGSPNeV in its sequences **(A)**. The size distribution of satGSPNeV-derived sRNAs in positive, negative, or both strands is in line with the normal distribution **(B)**. The 5′-nucleotide bias of satGSPNeV sRNAs **(C)**. Small RNA size distribution **(D)** and 5′-nucleotide bias **(E)** patterns of satGSPNeV are similar to those of other coinfecting RNA viruses; the color and histogram annotations are associated with abundance of specific sRNAs, with higher the histogram and deeper the red (or lighter the blue) representing higher the numbers of sRNAs.

Small RNAs of satGSPNeV spread almost evenly in the genome (SaCQ1-D) except for several hotspots (Figure 5A). Among satGSPNeV sRNAs, the most abundant were accumulated in the size range of 21 and 22 nt (Figure 5B), and in the 5′-nt of U and C (Figure 5C), similar to the characteristics for other viruses previously reported (Cao et al., 2019). The K–S tests showed that the size distributions, in the positive, negative or both strands, were in accordance with the normal (Gaussian) distribution when the predetermined significance level was 0.05, despite the shapes being different (Figure 5B). Clustering of available viruses and satellite in the CQ1-D, irrespective of the size (Figure 5D) or 5′-nt (Figure 5E), showed that satGSPNeV was closely associated with the GSPNeV RNA1 that encodes RNA-directed RNA polymerase (RdRp). Because intracellular replication of satellites relies intrinsically on proteins from their helper viruses (Palukaitis, 2017), it is not surprising that satellites, accompanied by helper viruses, may enter the same processes by host endogenous RNA silencing pathways onto the helper viruses, thereby resulting in similar small RNA patterns. Under similar selection pressures exerted by *Z. armatum* RNA silencing, accumulation of GSPNeV and satGSPNeV in the sample CQ1-D was still abundant, which may represent complex defense/counter-defense interactions between the hosts and viruses/satellite (Liu et al., 2017).

## Discussion

Over the last ten years, the FYD has become a restrictive factor in *Z. armatum* production in China after an endemic outbreak turned invasive and prevalent in the southwestern regions, including Chongqing Municipality, Yunnan, and Sichuan provinces, the main producing areas. A previous study revealed four different RNA viruses in one diseased tree (named CQ1-D in this study), and that GSPNeV was mainly associated with the FYD through field investigations (Cao et al., 2019). Here, to understand viral diversity of the FYD-affected trees by taking different sampling tissues (symptomatic vs. asymptomatic) and sites (five) into account, we adopted a more accurate HTS technique than conventional RT-PCR, whose detection range is limited for previous identified viral sequences. We conducted a large-scale survey, as 21 samples in total were analyzed. After a series of systematic data processes and molecular experiments, we newly identified a large, linear, single-stranded satellite RNA associated with GSPNeV, the satGSPNeV, and an ilarvirus, i.e., GSPIlV. They were taxonomically new based on comprehensive analyses and should be classified as a new species or satellite RNA under their own taxa, viral genus *Ilarvirus* and the subviral category, respectively (Briddon et al., 2012; Bujarski et al., 2019). SatGSPNeV was omitted in the previous analyses of CQ1-D (Cao et al., 2019), likely because of our annotation mistakes (contigs from the assembly of highly heterogeneous reads will sometimes be too short to be recognizable). As the current evidence showed, both GSPNeV and satGSPNeV were FYD-associated, while GSPIlV and the other viruses were not connected with the FYD and their symptoms on *Z. armatum* were still unclear due to the limited number of infected samples investigated. The relationships between these non-FYD-related viruses and the hosts, i.e., competition or mutualism, remain unknown.

In agroecosystems, plant viruses sometimes are destructive to crop industries because of the presence of sensitive cultivars (Moreno et al., 2008; Rybicki, 2015; Lefeuvre et al., 2019). In fact, observation of GSPNeV and satGSPNeV with high read proportions of the total reads sequenced from RNAs of symptomatic samples suggested their intense replication in the host. It is not difficult to imagine that they will compete with hosts for nutrient resources and may interfere with host normal physiological activity and cause visible symptoms (Ammara et al., 2017). Higher viral titers may also help the biological vectors perform more efficient viral acquisition and transmission (Gray et al., 1991), but not necessarily (Linak et al., 2020). Statically significant analyses disclosed the strong ability of GSPNeV and satGSPNeV to coinfect *Z. armatum*.

Genomic variation site analyses of the viruses and satellite allowed the assessment of their adaptability to different environments. Long- and short-term variation sites predicted from the comparisons of different consensus sequences and quasispecies, respectively, were largely discrepant in spite of a similar tendency in quantitative changes, suggesting the presence of circumstance-specific variations in the quasispecies. More strong evidence was obtained directly from observations of variable site patterns of the quasispecies from different locations. Nevertheless, we have to note that there are selective processes during viral evolution, so short-term variation sites are time-sensitive, changeable at different stages, and may not be ultimately preserved (García-Arenal et al., 2003). However, this can explain the aforementioned site discrepancies between the consensus sequences and quasispecies. When we calculated the nt-average variation sites, GSPNeV, satGSPNeV, and GSPEV had superior genetic variability in both quantity and evenness (more genomic regions) relative to other viruses, which may contribute to their competitive survival in hosts. More viral variation sites may increase the possibility of viral strains with better transmissibility and pathogenicity (severe vs. mild strains) (Niblett et al., 2000), despite a trade-off between this and the increasing deleterious mutations (Carrasco et al., 2007). As for genomic recombination, sequence variation brings about heterogeneous sequences that may recombine during replications in single cells, and this therefore makes the recombinations between divergent sequences predictable, even though recombinations will negatively regulate the divergences (Lai, 1992). In our data, higher sequence variations were associated with more genomic recombinations, probably because variable populations require recombinations for purging their deleterious mutations (Xiao et al., 2016). However, in this sense, the lower sequence variation and negative recombination prediction results of the GSPIdV RNAs do not mean the absence of recombinations; it is possible that these could not be predicted using a computer.

Cutting off of the transmission routes of viruses, if they do exist, is a simple but practical method for management and control before viruses lead to greater losses (Jones, 2006). Apart from genetic similarity at the both genomic ends, perfect consistency among GSPNeV RNAs and satGSPNeV in both phylogenetic and geographical relationships of the different isolates suggested their tight binding in both transmission and evolution, similar to other nepoviruses and their satellite RNAs (Alazem and Lin, 2017). Such a distinct distribution of different representative sequences, namely, homogeneous in each population and a lack of viral cross infections, may also suggest their weak natural movement ability and the involvement of human activities in transmission, such as seedling or plant material transportation. A similar situation was observed for GSPIdV RNAs, which, in previous work, were rarely detected in seedlings and fields (Cao et al., 2019). One possibility for the cooccurrence of GSPIdV with GSPNeV and satGSPNeV is that GSPIdV may benefit from this coinfection for easier invasion in the hosts, as synergism exists widely in plant viruses (Pruss et al., 1997; Mascia and Gallitelli, 2016). In contrast, it appears that the transmission of GSPNEV is more efficient than that of other viruses and satellites and naturally random, because more areas and heterogeneous sequences were involved and interrelated.

In summary, based on HTS-dependent viromic analyses, we discovered a new satellite RNA associated with GSPNeV and the FYD, as well as a new ilarvirus, and we studied their regional viral/subviral diversification, as well as that of other RNA viruses. This information can be useful for sustainable development of *Z. armatum* and helpful for understanding viral/subviral evolution. Then, our future works will have a shift of emphasis toward assessing biological properties of GSPNeV and satGSPNeV by construction of their infectious cDNA clones in planta. Meanwhile, parallel work is urgently needed, regarding elucidating the viral/subviral transmissions in fields, to restrain the dispersals.

## Data Availability Statement

The data presented in the study are deposited in the NCBI GenBank repository, accession numbers MH323432–MH323437 and MW962309–MW962391, and in the NCBI SRA repository, BioProject PRJNA732832, BioSample SAMN19341721–SAMN19341741, SRA accession numbers SRR14663372–SRR14663392.

## Author Contributions

MC conceived and designed the experiments. SZ, RL, XW, ZX, BZ, ZL, JZ, XD, ZT, SL, and YZ collected the samples and conducted the experiments. MC and SZ analyzed the data. MC, SZ, SL, and YZ discussed the results, drafted, and revised the manuscript. All authors read and approved the final draft of the manuscript.

## Conflict of Interest

The authors declare that the research was conducted in the absence of any commercial or financial relationships that could be construed as a potential conflict of interest.

## Footnotes

1
https://www.ncbi.nlm.nih.gov/orffinder/

2 https://www.ncbi.nlm.nih.gov/Structure/cdd/wrpsb.cgi

## Supplementary Material

Supplementary Figure 1 Detection of GSPNeV RNAs and satGSPNeV in an orchard affected by the flower yellowing disease. 1, 2, 4, 6, 8, 9, 11, symptomatic samples; 3, 5, 7, 10, 12, samples without symptoms; +, virus-positive control; H, healthy control; 0, water control.

Supplementary Figure 2 Comparisons of partial genomic terminal sequences from RNAs of green Sichuan pepper-ilarvirus (GSPIlV) suggested sequence conservation and the presence of complete coding sequences in the obtained sequences.

Supplementary Figure 3 Sequence comparisons of genomic RNAs of Sichuan pepper-ilarvirus (GSPIlV) with those of selected related viruses. Cucumber mosaic virus from the genus *Cucumovirus* was used as an outgroup. Three groups (G1, G2, G2) as subgroups under the genus *Ilarvirus* are indicated.

Supplementary Figure 4 Comparisons among GSPNeV RNAs and satGSPNeV showed some conserved sequences of the genomic ends.

Supplementary Figure 5 Matrixes of sequence identities between RNAs of different viral/subviral isolates obtained in this study.

Supplementary Figure 6 Correlations of numbers of variation sites from the consensus sequences and quasispecies of different viral/subviral RNAs and the common variation sites between the consensus sequences and quasispecies.

Supplementary Table 1 List of primers used in this study.
