# Supplementary figures and images for: Spatial Virome Analysis of Zanthoxylum armatum Trees Affected With the Flower Yellowing Disease
Source: Front Microbiol. 2021 Jun 28;12:702210. doi: 10.3389/fmicb.2021.702210 (PMC8298004; doi:10.3389/fmicb.2021.702210)

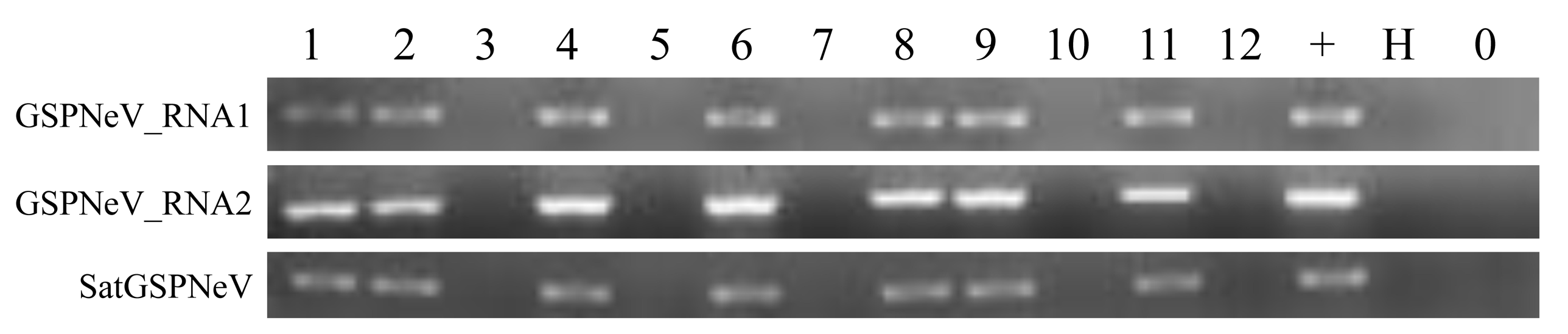

Supplement: Supplementary Figure 1 — Detection of GSPNeV RNAs and satGSPNeV in an orchard affected by the flower yellowing disease. 1, 2, 4, 6, 8, 9, 11, symptomatic samples; 3, 5, 7, 10, 12, samples without symptoms; +, virus-positive control; H, healthy control; 0, water control. [file Image_1.JPEG]

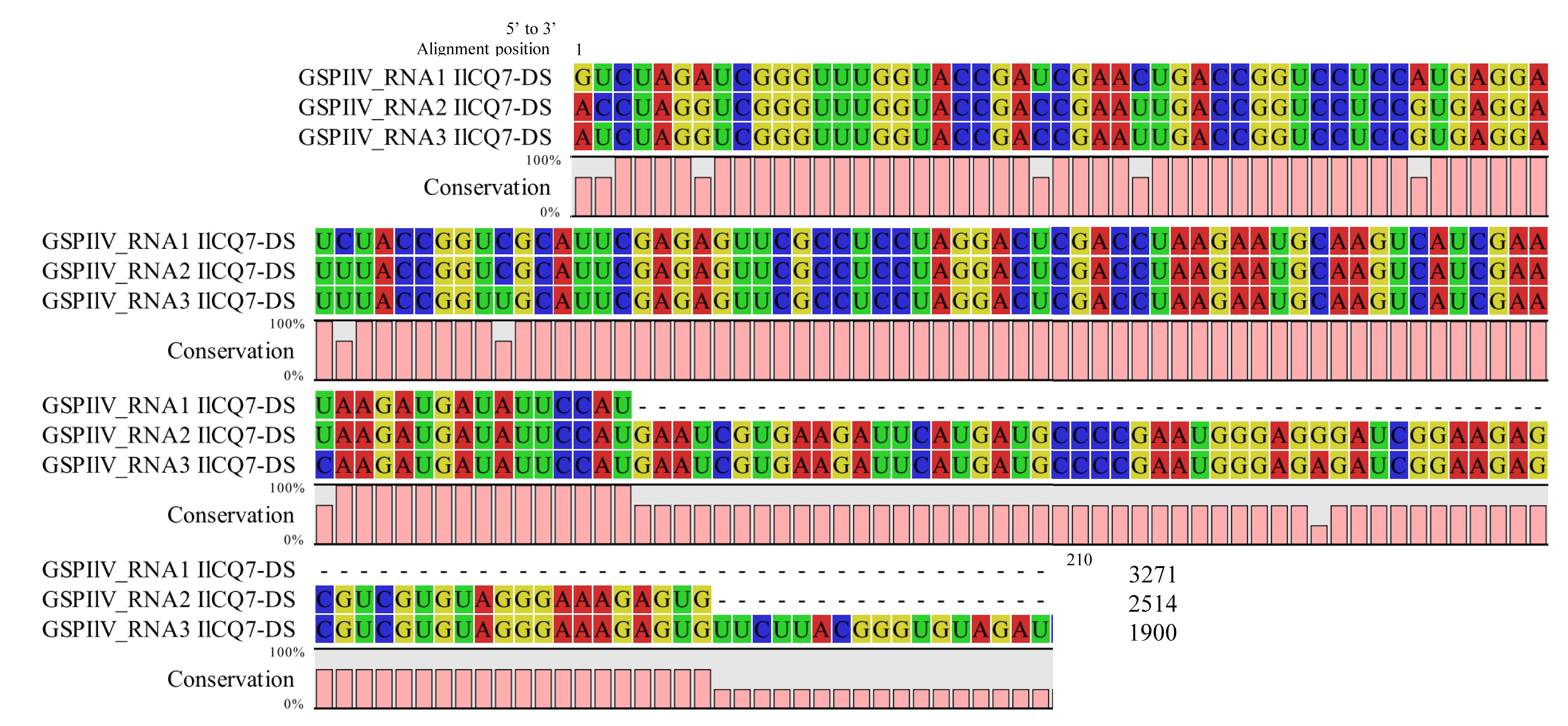

Supplement: Supplementary Figure 2 — Comparisons of partial genomic terminal sequences from RNAs of green Sichuan pepper-ilarvirus (GSPIlV) suggested sequence conservation and the presence of complete coding sequences in the obtained sequences. [file Image_2.JPEG]

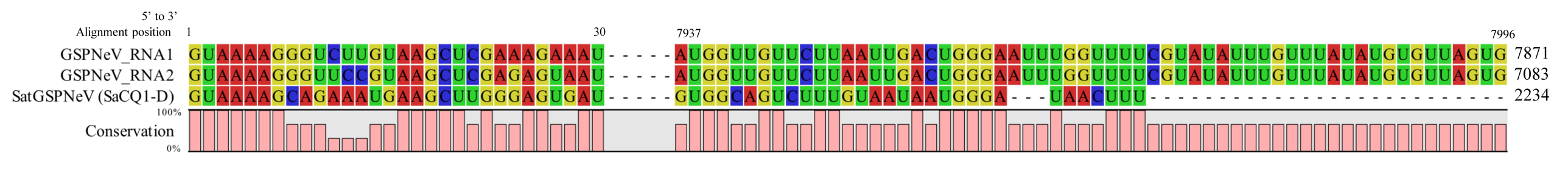

Supplement: Supplementary Figure 4 — Comparisons among GSPNeV RNAs and satGSPNeV showed some conserved sequences of the genomic ends. [file Image_4.JPEG]

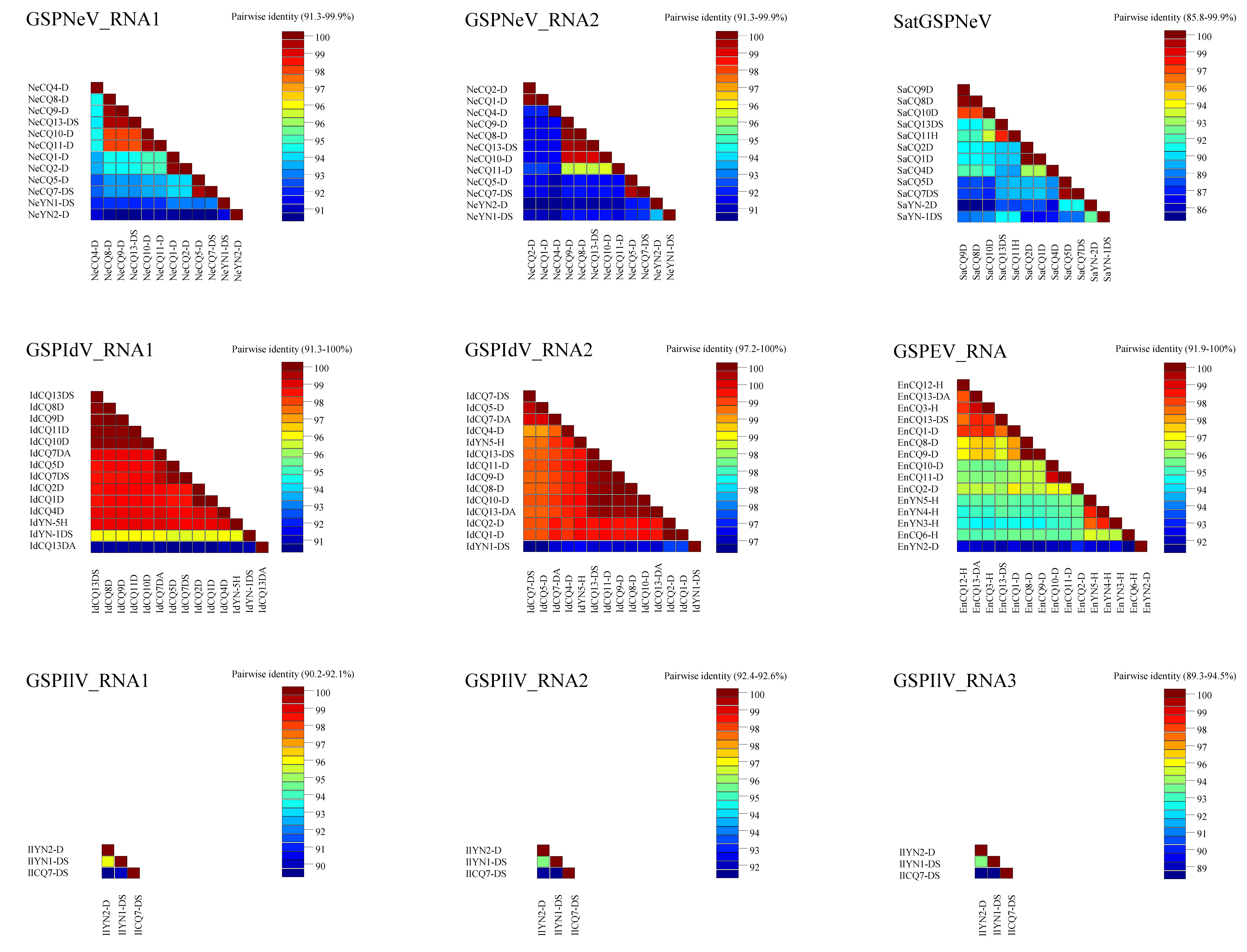

Supplement: Supplementary Figure 5 — Matrixes of sequence identities between RNAs of different viral/subviral isolates obtained in this study. [file Image_5.JPEG]

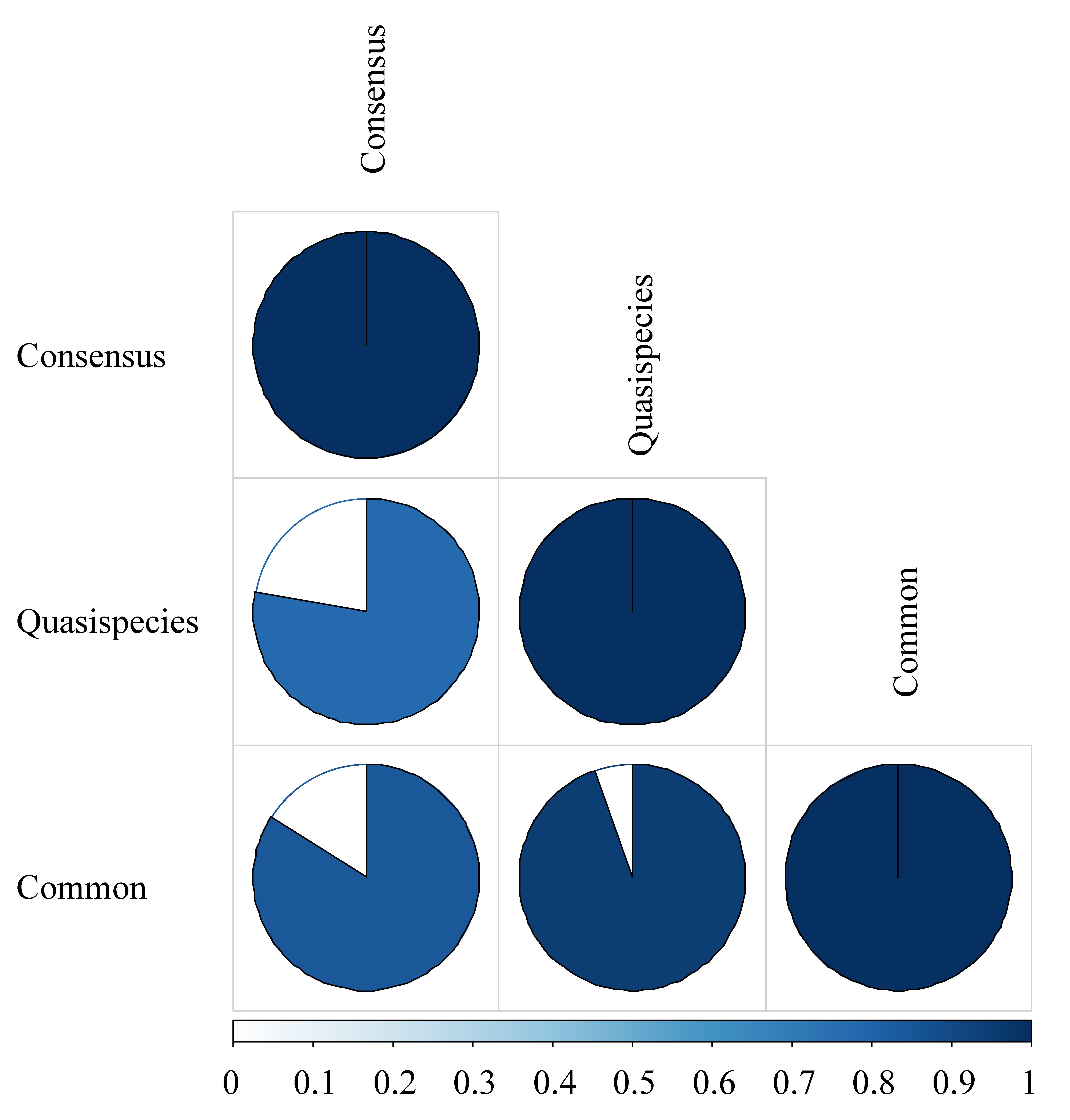

Supplement: Supplementary Figure 6 — Correlations of numbers of variation sites from the consensus sequences and quasispecies of different viral/subviral RNAs and the common variation sites between the consensus sequences and quasispecies. [file Image_6.JPEG]
